# Annotations

**Published:** 1898-08-27

**Authors:** 


### ANNOTATIONS.

### Hot Weather Precautions.

Most people, except those who are compelled to reside in the more crowded districts of our large towns, must have enjoyed the warmth and sunshine of the last few weeks. No doubt the heat has been great at times, but such heat as we experience in Great Britain need never cause serious discomfort. It is, however, of the first importance to remember that as the temperature goes up so it is desirable to pay attention to clothing, diet, and beverages. Alcohol should be avoided as far as possible; flannel is the best material to wear; and very little meat should be eaten. So far this year there has been no announcement of an outbreak of English cholera, but many people have no doubt suffered at least inconvenience from the absence of precautions in the matters referred to, milk which is not perfectly sweet, stone fruit (especially when stewed), and certain kinds of fish, like herrings, being very apt to produce sharp choleraic attacks. Attention to the simple precautions we have indicated will contribute to make the hot weather a season of joy and benefit to all who have sufficient intelligence to adopt them.

### Baby-Farming.

Is it not possible to regulate baby-farming better than at present? We are not yet within sight of a time when illegitimate children will no longer be born, nor a time when their mothers will not try to conceal their existence. Any attempt to force the public acknowledgment of an illegitimate child is certain to be a failure; but is it not possible to insist that no person shall take a child to bring up without having a certificate from the police or some responsible authority that she is fit for a position of so much trust and responsibility? A licence having been given, the holder of it should be under supervision, and be subject to occasional visits without notice from a doctor—by preference a woman doctor—appointed by the authorities. Anyone keeping a child other than their own, whether as adopted or simply as a boarder, without such a licence, should be liable to a penalty for this of itself, even if the child were well enough taken care of. There need be no disgrace in keeping a baby-farm; it is a way in which many a decent poor woman might earn a living, and might often be kinder to a poor little "love-child" than its own mother, to whom it would be a constant reminder of her own frailty; but at present it is a disreputable occupation, taken up by women of doubtful character and little humanity, who are as often as not in tacit league with the child's nearest friends to put it out of the world as soon as possible. When too many children die under one roof there may be an inquiry, and the baby-farmer be punished, but it seems difficult to put an end to the evils of the system. It is, indeed, difficult to make provision for those hapless children without encouraging immorality, but the punishment of anyone who keeps a foster-child without permission, whether the child is treated well or ill, would tend to the elimination of that class of woman who takes a child with a £10 note on the assurance that there will be "no after claim," as it is phrased—no inquiry in the case of death would be more like the truth. It is something to punish the cruel baby-farmer after her nurslings are dead; but in the meantime the children are dead, and humanity demands complete protection for the living infants against the slow starvation and neglect which are the baby-farmer's weapons.

### Contemptible Fees!

A writer has lately asserted that the acceptance of "contemptible fees" for responsible professional service is "degrading to the individual and derogatory to the dignity of a learned profession." This is a very noble sentiment, and would sound well in an after-dinner speech or an inaugural address; but when we look at it in calmer moments we have to ask whether it fits in with the everyday experience of the working world in which we live. What is a proper fee? To this apparently simple question no general answer can be given. Sometimes—and this is where the rub comes—it must be very low indeed, unless we are prepared to hand over the whole of the medical attendance on the working-classes to charitable agencies, but it need not therefore be "degrading." It is not easy to put the matter into figures, but, from all the accounts one hears of the amount of work which is done by the poor-law medical officers, it seems fair to believe that they are the worst paid of all. Yet poor-law appointments are held by honourable and honoured members of our profession in every part of the country. It will not do, then, to admit that the acceptance of "contemptible fees" is degrading. What the profession wants is a system by which these small fees can be collected without loss—loss of money, loss of time, and loss of temper; and this, we fear, can only be obtained by some adaptation of the provident system. We say "we fear" because we know that the provident system stinks in the nostrils of the profession, and that it is the disinclination which is felt by many well-meaning medical men to do anything which shall in any way encourage an extension of this system which is one of the stumbling-blocks in the way of obtaining proper remuneration for professional services rendered to the labouring classes. What, however, is clear to all who think and who observe is that the great mass of the inhabitants of these islands are paid weekly or fortnightly wages, and that of these the great mass spend their wages as they come. These people are often capitalists in their own way, that is, by belonging to friendly societies and sick clubs, by which they are secured from want; but they are not capitalists in the sense of being able to obtain solid money with which to pay bills, and so long as the medical profession cannot devise a better system of obtaining payment than that of sending in bills they are not likely to obtain satisfactory remuneration for their attendance on the labouring classes. If we are to be relieved from what is said to be degrading and derogatory in the acceptance of small fees the provident system must be accepted in its entirety, and must be organised and worked by the doctors themselves. There ought to be no difficulty whatever about it if medical men in each district would but take the trouble to combine, but whatever is done its organisation must not be allowed to fall into non-medical hands.

